# *Cryptococcus deuterogattii* Lung Infection Leads to a Prothrombotic State Revealed by Proteomics and Functional Analysis

**DOI:** 10.1021/acsomega.6c07029

**Published:** 2026-10-28

**Authors:** Laura Rascovetzki Saciloto-de-Oliveira, Markus Berger, Luana Cardozo, Camila Innocente-Alves, David Driemeier, John R. Yates, Marilene Henning Vainstein, Jorge Almeida Guimarães, Lucélia Santi, Walter Orlando Beys-da-Silva

**Affiliations:** † Laboratório de Microbiologia Molecular e Proteômica, Faculdade de Farmácia, Universidade Federal do Rio Grande do Sul (UFRGS), Porto Alegre, RS 90610-000, Brazil; ‡ Faculdade de Farmácia, Universidade Federal do Rio Grande do Sul (UFRGS), Porto Alegre, RS 90610-000, Brazil; § Laboratório de Neuroproteômica, Universidade de Campinas (Unicamp), Campinas, SP 13083-970, Brazil; ∥ Departamento de Patologia Clínica Veterinária, Faculdade de Medicina Veterinária, Universidade Federal do Rio Grande do Sul (UFRGS), Porto Alegre, RS 91540-000, Brazil; ⊥ Department of Molecular Medicine, Scripps Research, La Jolla, California 92037, United States; # Centro de Biotecnologia, Universidade Federal do Rio Grande do Sul (UFRGS), Porto Alegre, RS 90040-060, Brazil

## Abstract

Cryptococcosis caused by *Cryptococcus deuterogattii* is a life-threatening fungal infection increasingly reported in immunocompetent individuals. Although clinical evidence suggests that invasive fungal diseases could impact coagulation, the molecular mechanisms linking cryptococcal infection to hemostatic dysregulation remain unexplored. In this study, we employed an integrative approach combining *in vivo* rat lung infection model, proteomics, and *in vitro* assays to investigate alterations in coagulation and hemostasis induced by *C. deuterogattii* R265. Morphological and histological evaluations revealed progressive lung damage and cryptococcoma formation. Functional assays showed early thrombocytopenia, increased thrombin activity, reduced prothrombin time, and enhanced platelet aggregation, indicating a procoagulant state in the infection. Proteomic analysis identified the upregulation of platelet activation pathways and key prothrombotic proteins at 3 days post-inoculation (dpi), along with downregulation of anticoagulant regulators such as thrombomodulin and antithrombin III. At 14 dpi, a widespread protein downregulation suggests systemic collapse of host functions. Data are available via ProteomeXchange with identifier PXD077765. Coculture of A7R5 cells with *C. deuterogattii* confirmed increased tissue factor expression, reinforcing the role of the fungus in triggering thrombo-inflammatory responses and validating proteomics data. Our results provide the first molecular evidence linking cryptococcal infection to a dysregulated hemostatic profile, shedding light on host–pathogen interaction and suggesting that targeting coagulation pathways may offer novel therapeutic avenues in cryptococcosis management.

## Graphical abstract

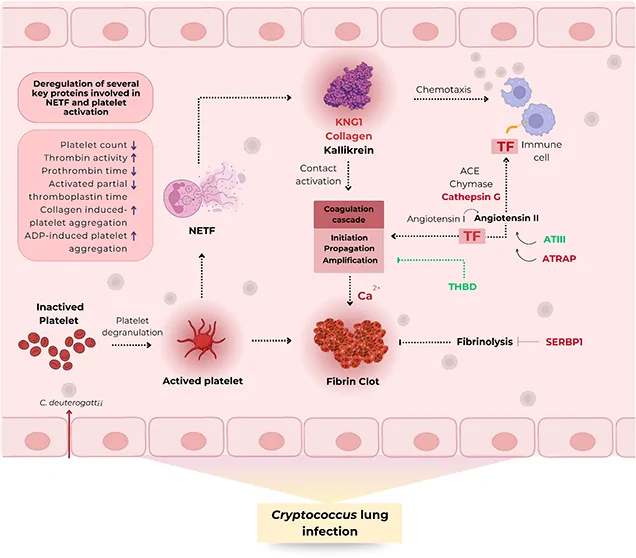

## Introduction

Cryptococcosis, a disease caused by *Cryptococcus* species complexes, is considered one of the most common invasive fungal diseases (IFDs) in humans and is estimated to kill more than 180,000 people worldwide.^1–3^ Among yeasts of the *Cryptococcus* genus, *Cryptococcus gattii* and *Cryptococcus neoformans* species complex are considered fungal priority pathogens by the World Health Organization (WHO).^4^

The *C. gattii* species complex is usually more virulent than the *C. neoformans* species complex and can infect both immunocompetent and immunosuppressed individuals.^5,6^ In immunocompetent individuals, the clinical presentation includes productive cough, pleuritic pain, dyspnea, hemoptysis, and weight loss.^7^ Cryptococcosis is most common in tropical and subtropical regions.^5,6^ However, between 1999 and 2004, outbreaks of human cryptococcosis caused by *Cryptococcus deuterogattii*, a fungus belonging to the *C. gattii* complex, occurred in unexpected regions such as Vancouver Island, Canada, and throughout the US Pacific Northwest and California, and since then, there has been an increase in the number of *C. gattii* complex infections worldwide.^5,8^

Although host immunity helps to fight fungal pathogens, dysregulation of related responses can have a deleterious effect on the host itself. The activation of the inflammatory response in the face of invasion not only plays a fundamental role in the body’s defense but also directly influences hemostasis and coagulation, processes that help contain the spread of the infectious agent.^9,10^ The direct impact of coagulation on the inflammatory response may be noticed by the relationship between thrombin and macrophages, as it has been shown that thrombin induces the polarization of macrophages toward a pro-inflammatory phenotype.^10^ Furthermore, another link between these processes is centered on tissue factor (TF), which plays a central role in the activation of coagulation, acts as an inflammatory modulator, and its increased expression during infections favors the occurrence of thrombotic events.^11,12^ Finally, inflammation can also compromise natural anticoagulant pathways, leading to a state of hypercoagulability, which further highlights the interdependence between the inflammatory and hemostatic systems.^9^

Several studies have employed proteomics approaches to study fungal infections, but most have focused on fungal molecular changes, while fewer papers address host molecular changes.^13^ In this context, considering the relationship between coagulation processes, the infectious process discussed above, the impact of cryptococcosis worldwide, and the importance of studying infection from the host perspective, here we investigated, through molecular analysis of proteomic data, the impact of cryptococcosis on hemostasis and blood coagulation in experimentally infected rats. Our findings provide the first molecular evidence linking cryptococcal infection to hemostatic dysregulation, including the upregulation of platelet activation pathways and key prothrombotic proteins. Additionally, a prothrombotic profile was confirmed by in vitro functional assays.

## Results

### *In Vivo* Experiments

#### Morphological Analysis

Infected rats exhibited clear signs of acute illness after 3 dpi, including progressive weakness, fatigue, prostration, labored breathing, increased heart rate, and raised fur (piloerection). At 14 dpi, the rats presented a significant worsening of clinical presentation. The animals in the control group exhibited no adverse effects. Yet, R265-inoculated rats (3 dpi and 14 dpi) post-mortem examination showed extensive lung damage with fibrotic tissue and cryptococcomas formation when compared with noninfected animals that presented normal lung morphology. Accordingly, at 3 dpi, cryptococcomas were dark gray, and at 14 dpi, they became light gray (Figure 1A). Confirming these observations, histological sections of the control animals showed no alterations (not shown). In the sections of inoculated rats, *C. deuterogattii* was observed to disseminate from the pulmonary parenchyma and compromise the pulmonary parenchyma and bronchioles (Figure 1B). Additionally, it illustrates these histological findings, specifically detailing the invasion of a pulmonary blood vessel by *C. deuterogattii* yeast cells and the associated inflammatory perivascular mononuclear infiltration (Figure 1C).

**Figure 1:**
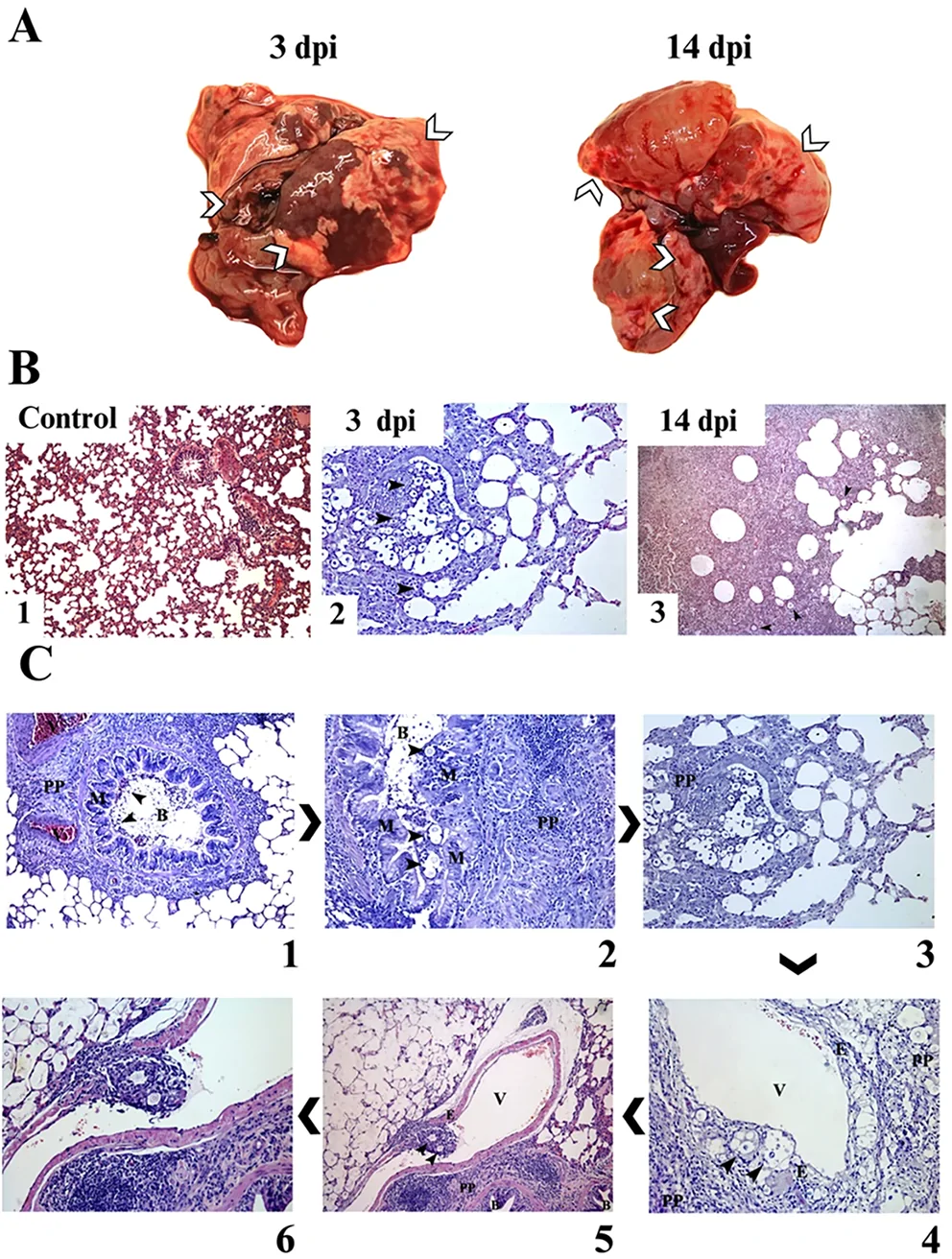
Morphological and histological analysis. (A) Cryptococcomas (white arrow) in rat lungs at 3 and 14 dpi, showing enlarged lungs with rounded edges and grayish spots of pneumonia. At 3 dpi, they were dark gray, and at 14 dpi, they became light gray. (B, C) Histological sections of rat lungs infected with the R265 strain. (B1) Control rat lung showing preserved pulmonary architecture (no inoculation) (10×). (B2) Dissemination of the R265 strain to the pulmonary parenchyma (black arrows, 20×). (B3) Compromise of the pulmonary parenchyma and bronchiole by the R265 strain (black arrow, 10×). (C) *C. deuterogattii* R265 cells invading rat lung blood vessel. (C1–C3) Entry of *C. deuterogattii* cells and the histiocytic inflammatory involvement in the bronchioles (B) and surrounding lung parenchyma (PP) (10×). (C4–C6) *C. deuterogattii* yeast cells (black arrow) invading a blood vessel (V), accompanied by inflammatory perivascular mononuclear histiocytic infiltration extending to the perivascular tissue (PP) (10×). M: mucosa; E: vascular endothelium.

#### Platelet Aggregation and Coagulation Parameters of *C. deuterogattii*-Infected Rats

Platelet aggregation and coagulation parameters were monitored in rats infected intranasally with *C. deuterogatti* strain R265. The parameters indicated a procoagulant state in the early days of infection followed by changes in platelet aggregation. On the third day post-infection, a slight reduction in platelet count was observed. Additionally, recalcification and activated partial thromboplastin time (aPTT) decreased. Thrombin activity increased during this period. At the same time, the prothrombin time was significantly reduced. On the 14th day, there was significant platelet reduction. Yet, adenosine diphosphate (ADP)- and collagen-induced platelet aggregation significantly increased compared with the control. Thrombin activity remained elevated, contributing to the persistence of the procoagulant state. Prothrombin time was still reduced, although with a slight recovery compared to the third day. Meanwhile, recalcification time and aPTT returned to values close to those of the control group (Figure 2).

**Figure 2:**
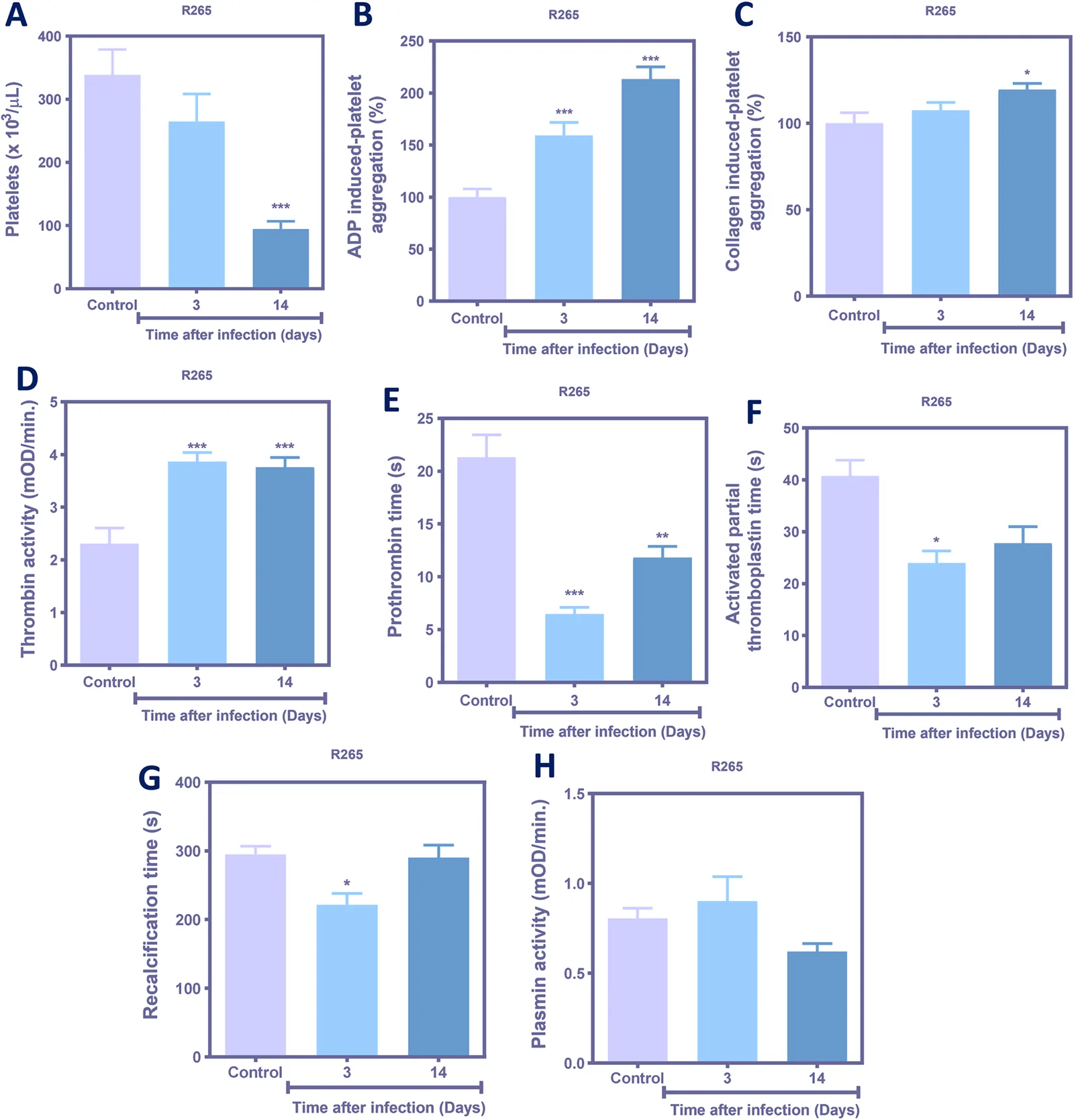
Platelet aggregation and coagulation parameters of Wistar rats infected with *C. deuterogattii* R265. (A) Platelet count at 3 and 14 dpi; (B) ADP-induced platelet aggregation in 3 and 14 dpi; (C) collagen-induced platelet aggregation in 3 and 14 dpi; (D) thrombin activity in 3 and 14 dpi; (E) prothrombin time in 3 and 14 dpi; (F) activated partial thromboplastin time in 3 and 14 dpi; (G) recalcification time in 3 and 14 dpi; (H) plasmin activity in 3 and 14 dpi. One-way ANOVA followed by Bonferroni post-test was used as a hypothesis test. Statistical difference was considered at *p < 0.05, **p < 0.01, and ***p < 0.001.

### Proteomic Analysis

#### Differential Proteome Overview

The mass spectrometry proteomics data have been deposited in the ProteomeXchange Consortium via the PRIDE partner repository with the data set identifier PXD077765. We analyzed two different time points of infection compared to control: proteins identified in rat lungs at 3 and 14 dpi (Figure 3A,B). A total of 1032 proteins were identified in the 3 dpi group, with 405 shared with the control. Among these common proteins, we focused on differentially expressed proteins, with 10 found upregulated and 63 downregulated in the 3 dpi group. In the 14 dpi group, 245 proteins were identified, with 166 shared with the control. Among the common proteins, 2 were found upregulated and 18 downregulated (Tables S1 and S2).

**Figure 3:**
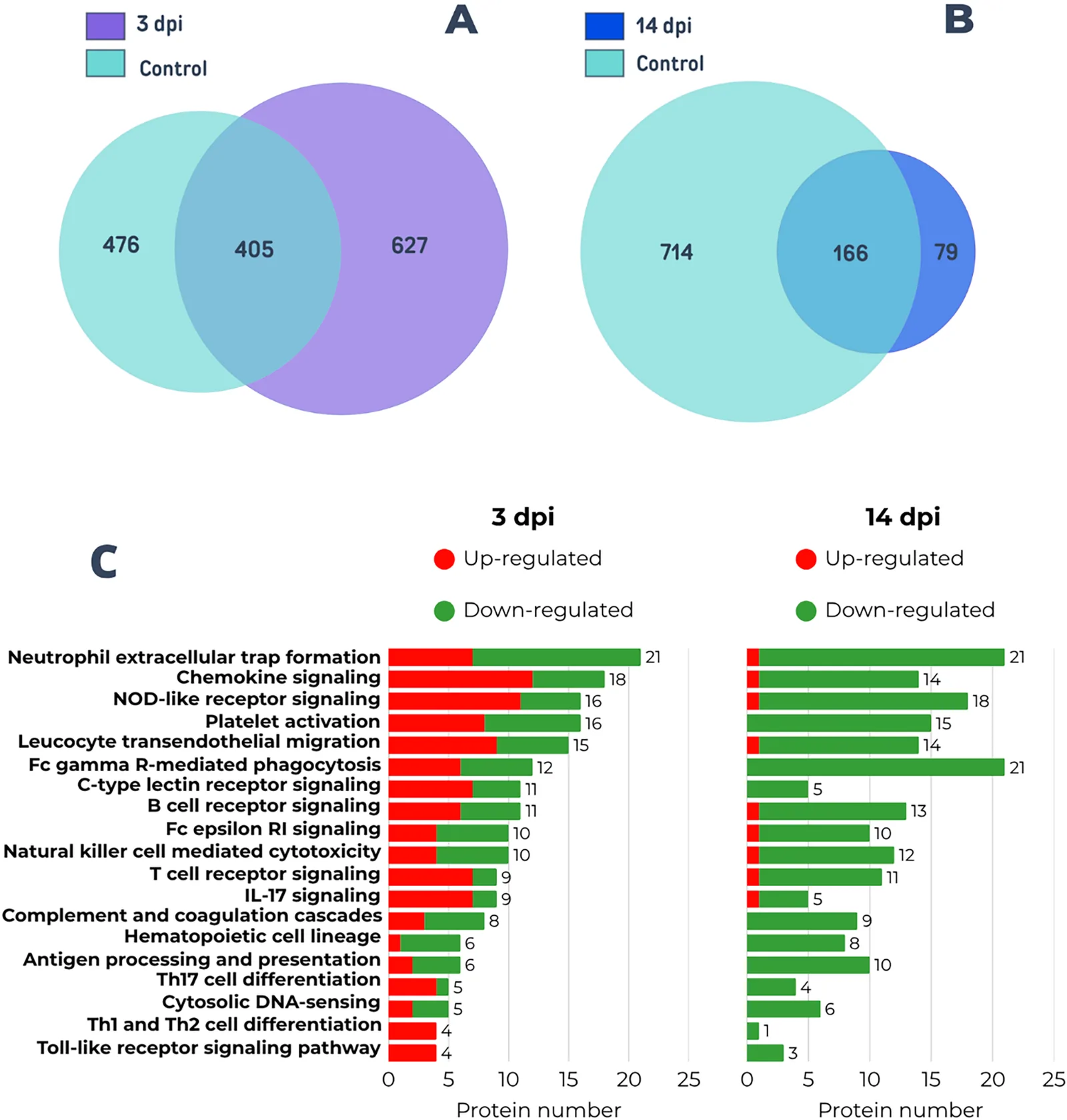
Venn diagrams (A, B) show the distribution and overlap of proteins identified in rat lungs infected with *Cryptococcus deuterogattii* R265 at 3 (A) and 14 (B) dpi, compared to uninfected controls. Protein identification was performed using the AAPV module of PatternLab. (C) Summary of differentially regulated KEGG immune system pathways at both time points, indicating the number of upregulated (red) and downregulated (green) proteins. For each pathway, the left bar corresponds to 3 dpi and the right bar to 14 dpi.

#### Functional Analysis

Gene ontology (GO) enrichment analysis results at 3 dpi showed a predominance of proteins related to the tricarboxylic acid cycle, response to hydrogen peroxide, positive regulation of translation, actin cytoskeleton organization, and actin filament organization at the biological process level. Additionally, the most abundant molecular function at 3 dpi was SH3 domain binding, and the phagocytic cup was the most abundant cellular component. At 14 dpi, biological process classification, proteins related to positive regulation of lamellipodium assembly, tricarboxylic acid cycle, and positive regulation of phagocytosis were predominant. In addition, the most abundant molecular function was aminopeptidase activity, and the vesicle membrane was the predominant cellular component (Figure S1).

Regarding immune system pathways altered due to the differential expression of proteins in infection, 19 pathways were identified. The higher number of categorized proteins in the 3 dpi group was linked to neutrophil extracellular trap formation (NETF), followed by the chemokine signaling pathway, NOD-like receptor signaling pathway, platelet activation, and leukocyte transendothelial migration. In the 14 dpi group, it is possible to highlight the highest one, together with NETF, the FC γR-mediated phagocytosis (Figure 3C).

#### Gene Set Enrichment Analysis (GSEA)

According to the normalized enrichment score (NES), the most activated pathways at 3 dpi were platelet degranulation, response to elevated platelet cytosolic Ca^2+^, interleukin-12 stimulation, gene and protein expression by JAK-STAT signaling after interleukin-12 stimulation, hemostasis, and neutrophil degranulation. On the other hand, the NES score shows a potential deactivation of pathways, such as those related to energetic metabolism, biological oxidation, and the role of phospholipids in phagocytosis (Figure 4A).

**Figure 4:**
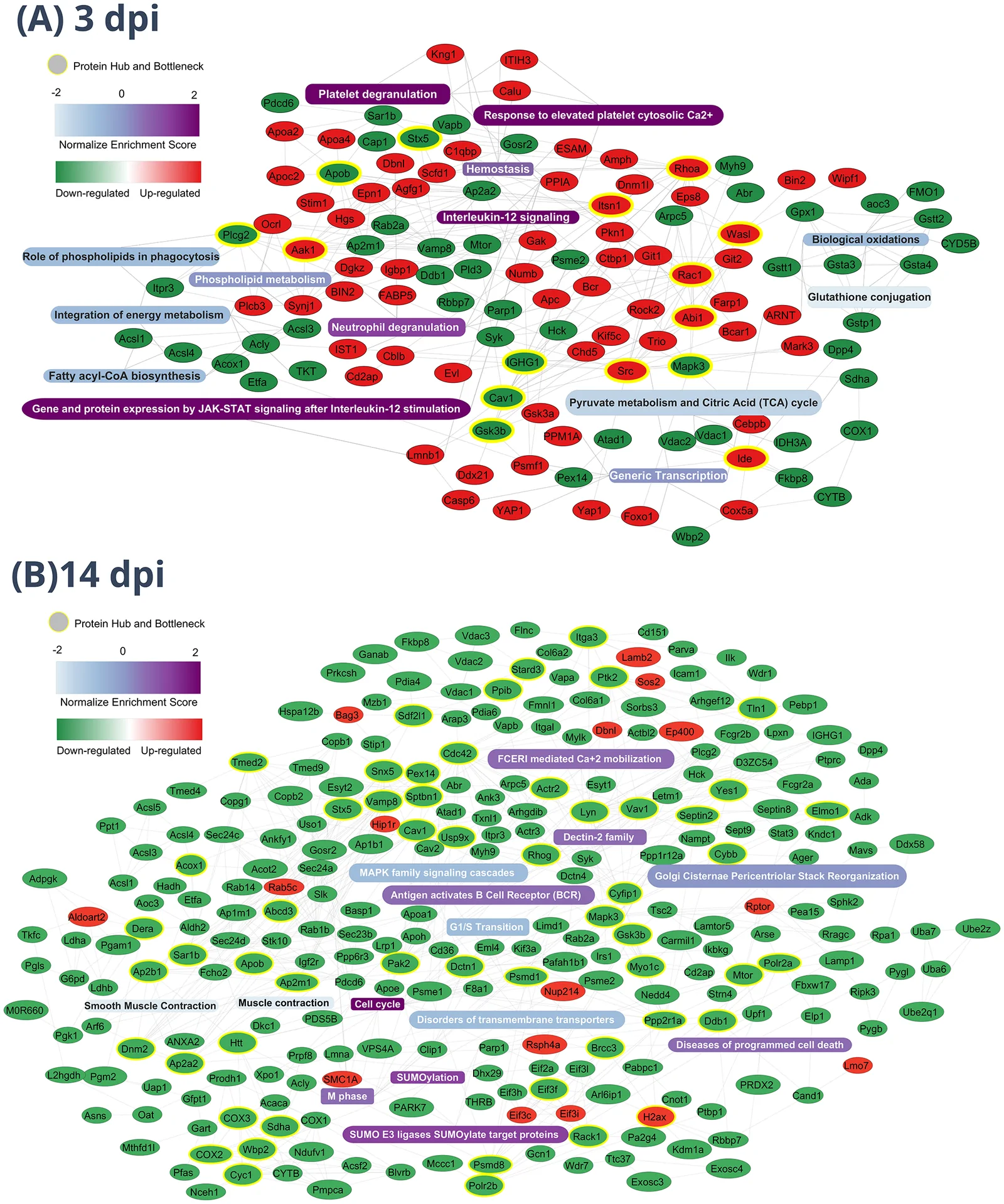
Network of GSEA of proteins identified in the 3 dpi (A) and 14 dpi (B) groups. Significant GSEA clusters/pathways are presented in ellipse form (blue to purple in color scale), and proteins (represented by their UniProt Entry name) upregulated (red) or downregulated (green) are in circles. The top 10 gene sets for the normalized enrichment score (NES) were considered significantly enriched. NES > 0 suggests activated pathways, and NES < 0 suggests inactivated pathways. The network was constructed using Cytoscape 3.10.2 software and the STRING database. The betweenness and degree analysis was performed using RStudio packages Rcy3 and Igraph. Note that the network represents a subset of the total proteins identified in the proteomic data set, as only proteins meeting the enrichment and connectivity thresholds were included.

At 14 dpi, most proteins were downregulated or control-exclusive, but GSEA shows an enrichment of pathways related to cell cycle and SUMOylation, among others (Figure 4B).

#### Tissue Factor (TF) Expression

Compared to the control group (CT), which was not exposed to the fungus, TF protein expression increased significantly after 3 and 12 h of coculture, as indicated by densitometric analysis normalized to β-actin. Although a slight increase in TF expression was observed at 6 h, this change was not statistically significant. The immunoblotting image supports these findings, showing more intense TF bands at 3 and 12 h, while β-actin bands remained consistent across all samples, confirming equal protein loading (Figure 5).

**Figure 5:**
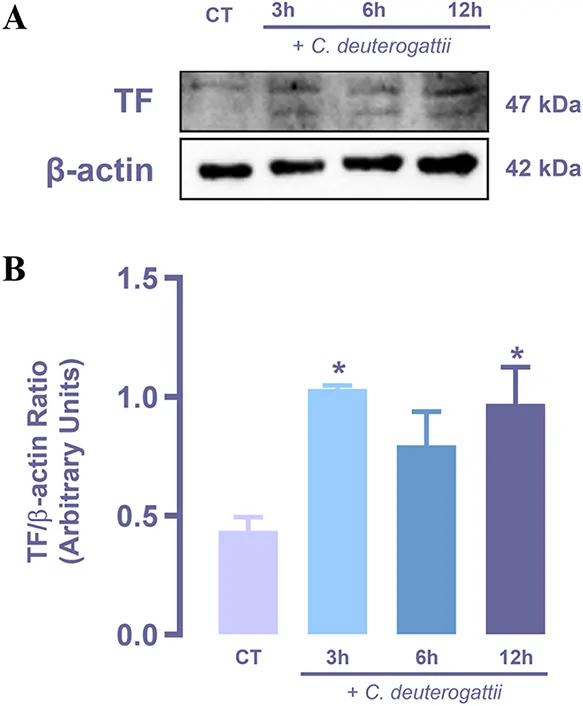
Tissue factor (TF) expression in vascular smooth muscle cells (VSMCs) cocultured with *C. deuterogattii* R265 for 3, 6, and 12 h. (A) Bands visualized on the iBright Imaging System. (B) Representative Western blot and densitometric analysis of TF normalized to β-actin. Significance was determined using one-way ANOVA, *p < 0.05.

## Discussion

Understanding the molecular mechanisms underlying *Cryptococcus* pathogenesis is crucial to improving clinical outcomes and survival rates.^14–16^ In this context, we investigated the molecular and biochemical impact of *C. deuterogattii* R265 strain infection in rat lungs. Accordingly, infected rats displayed a prothrombotic profile, with a significant decrease in platelet count from 3 dpi, likely due to increased consumption from coagulation activation.^17,18^ Platelet hyperreactivity was also evident as ADP- and collagen-induced aggregation significantly increased at 3 and 14 dpi. Correlating these findings with proteomics helps elucidate the molecular infection mechanisms.

Proteomic enrichment at 3 dpi revealed coordinated alterations in platelet activation, hemostasis, inflammatory signaling, and leukocyte–endothelial interactions. The enrichment of NET formation and platelet-related pathways is particularly relevant because NETs can provide a scaffold for thrombus formation, linking innate immune activation to the prothrombotic phenotype observed here.^19,20^ Concerning their roles in coagulation and hemostasis, platelets can also regulate immune cells such as neutrophils, macrophages, and monocytes.^21^ In addition, platelet activation during infection-related inflammation can trigger thrombotic events, worsening clinical outcomes.^22,23^

At 3 dpi, increased abundance of proteins involved in platelet activation and cytoskeletal remodeling, including Transforming protein RhoA (RhoA), Rho-associated protein kinase 2 (ROCK2), Proto-oncogene tyrosine-protein kinase Src (SRC), and Stromal interaction molecule 1 (STIM1), was consistent with enhanced platelet reactivity observed experimentally.^24–26^ Changes in integrin β-1 (ITB1) and collagen α-1­(I) chain (CO1A1) (exclusively identified at 3 dpi) further suggest alterations in cell–matrix interactions,^26,27^ while the exclusive detection of synaptosomal-associated protein 23 (SNAP23), vesicle-associated membrane protein 8 (VAMP8), and Rho guanine nucleotide exchange factor 12 (ARHGEF12) in controls may indicate reduced detectable abundance of proteins involved in platelet granule exocytosis, which is crucial for maintaining vascular integrity and preventing excessive thrombosis..^28–31^ Yet, the disruption of vascular homeostasis is a known critical step in cryptococcal pathogenesis. Recent studies demonstrate that host immune factors, such as IL-6, are pivotal in maintaining endothelial barrier integrity to prevent fungal dissemination, reinforcing the relevance of the vascular alterations identified in our pulmonary model.^32^

Another interesting proteomic finding was the exclusive identification of Thrombospondin-1 (TSP1) at 3 dpi (Table S1). This hemostatic mediator promotes platelet activation by inhibiting cAMP signaling^33^ and interacts with factor C5a to enhance chemotaxis,^34^ while also blocking cGMP–protein kinase activation, thus fostering platelet aggregation and adhesion,^35^ consistent with *in vivo* observations. Beyond hemostasis, TSP1 inhibits angiogenesis by binding CD36 on endothelial cells, inducing apoptosis and suppressing new vessel formation,^36–39^ potentially relevant in prothrombotic, inflammatory states with impaired vascular remodeling. Additionally, TSP1 could interfere with nitric oxide (NO) signaling, lowering its bioavailability and promoting vasoconstriction, and can interact with leukocytes to activate macrophages, reinforcing its role in thrombo-inflammatory progression.^35–38^

In parallel, recalcification time and activated partial thromboplastin time (APTT) showed a reduction in infected animals at 3 dpi, suggesting early activation of the coagulation cascade.^39^ In concordance, we exclusively identified at 3 dpi the kininogen-1 protein (KNG1). This protein acts as a cofactor in the activation of factor XII, initiating the contact activation pathway of coagulation and contributing to thrombin and fibrin formation.^40^ Additionally, this protein plays a fundamental role in the inflammatory response, acting as a precursor of bradykinin, a potent vasodilator, and an inflammatory mediator.^41^

Additionally, the type-1 angiotensin II receptor-associated protein (ATRAP) was exclusively identified at 3 dpi. This protein acts as a negative regulator of the type-1 angiotensin II receptor (AT1R), inducing its internalization and reducing its signaling.^42^ Indeed, transfection of ATRAP potentiated AT1R internalization in vascular smooth muscle cells after Ang II stimulation.^43^ Interestingly, Ang II may be converted by cathepsin G,^44^ which was found exclusively at 3 dpi, suggesting the expression of Ang II. Yet, antithrombin III (ATIII) was identified exclusively in the control, and this protein is described as effectively preventing Ang II-mediated accelerated thrombosis and acts as an inhibitory component in thrombin formation and as an inhibitor of factor IXa and factor Xa.^45^

Other than vasoconstriction, Ang II also stimulates TF,^46^ which was found to be increased in A7R5 cells cocultured with *C. deuterogattii* R265 after 3 and 12 h. TF is a transmembrane glycoprotein that acts as an initiator of the extrinsic pathway, forming a complex with factor VIIa, which leads to the formation of thrombin and subsequently clot formation. In addition to its role in coagulation, TF is also involved in the inflammatory response, modulating the activation of immune cells and promoting the release of pro-inflammatory cytokines. Its increased expression under pathological conditions, such as infections and cardiovascular diseases, contributes to a state of hypercoagulability associated with inflammation.^47,48^ Indeed, increased TF validates the proteomic data, reinforcing that *C. deuterogattii* promotes an environment favorable to thrombotic events that could be harmful to the infected host.

Moreover, thrombin activity was also significantly elevated in infected rats at 3 and 14 dpi. Interestingly, in proteomic data, we identified thrombomodulin (THBD) only under the control condition. This protein plays a fundamental role in coagulation regulation and an inflammatory response. In addition to inactivating thrombin by forming a complex with it, THBD activates protein C, which exerts anticoagulant effects by inhibiting factors Va and VIIIa.^49–56^ In systemic infections, such as sepsis, reduced THBD levels in the endothelium may contribute to disseminated intravascular coagulation (DIC), a severe condition characterized by uncontrolled thrombus formation.^55,56^

The conversion of the inactive precursor plasminogen into the active serine protease plasmin, which breaks down fibrin within thrombi, represents a critical event in thrombolysis.^57^ In infected rats, plasmin activity did not show significant changes, which may indicate that fibrinolysis does not proportionally accompany coagulation activation, increasing the risk of thrombotic events. Accordingly, plasminogen activator inhibitor 1 RNA-binding protein (SERBP1) was identified as being exclusive at 3 dpi. This protein is involved in the post-transcriptional regulation of plasminogen activator inhibitor 1 (PAI-1), especially through interaction with the 3′UTR region of mRNA, affecting its stability and, consequently, the amount of PAI-1 produced.^58–60^ Thus, the expression of SERBP1 at 3 dpi can be indicative of positive regulation of PAI-1, contributing to the coagulative profile observed in infected rats. Additionally, plasminogen was found to be downregulated at 14 dpi, which is suggestive of a lower level of expression of this protein, consistent with the prothrombotic profile observed. Finally, prothrombin time (PT) showed a marked reduction on the third day with a slight increase on the 14th day, suggesting rapid clot formation during this infection stage.

In conclusion, rats infected with *C. deuterogattii* R265 exhibit molecular and biochemical prothrombotic alterations within 3 dpi. Conversely, lungs from infected rats at 14 dpi show an expressive downregulation of most proteins. This remarkable decrease in protein expression suggests a potential systemic collapse in biochemical pathways, possibly reflecting a near-terminal state in which the host’s ability to maintain essential cellular functions is severely compromised. Taken together, our findings indicate that *C. deuterogattii* infection induces significant alterations in coagulation and hemostasis, contributing to a prothrombotic state. The observed dysregulation in platelet activation, inflammatory pathways, and the renin-angiotensin system suggests that cryptococcosis may promote vascular complications that exacerbate disease severity. Additionally, our proteomics findings are in concordance with clinical signals identified in infected rats and coculture with A7R5 cells.

These findings highlight the importance of further investigating host–pathogen interactions in cryptococcosis and provide experimental data that support the need for the monitoring of coagulation parameters in patients with cryptococcosis. These findings also raise the question of whether existing anticoagulant or antiplatelet therapies could influence thrombo-inflammatory processes during cryptococcosis. However, their potential effects remain unknown, and clinical studies are needed before any therapeutic implications can be drawn. Furthermore, understanding these specific host–pathogen interactions could complement ongoing efforts to develop effective immunotherapies and vaccines against this pathogen.^61,62^

It is important to mention that this study has some limitations that should be considered in interpreting the findings. First, the use of a rat model limits the direct translation of these results to human cryptococcosis. Second, the study focused on the highly virulent and clinically relevant *C. deuterogattii* R265 strain. Therefore, further studies are needed to determine whether the observed hemostatic alterations also occur with other *C. deuterogattii* strains or represent a broader response to severe fungal infections. Finally, although the proteomic findings were supported by functional assays demonstrating alterations in coagulation and hemostasis, the individual candidate proteins were not comprehensively validated. Future studies in clinical samples and with additional strains will be important to further establish the translational relevance and specificity of these findings. Despite these limitations, the results described here may help elucidate the molecular mechanisms of cryptococcosis, revealing previously undocumented features of the infection. Such mechanisms and pathways are illustrated in Figure 6.

**Figure 6:**
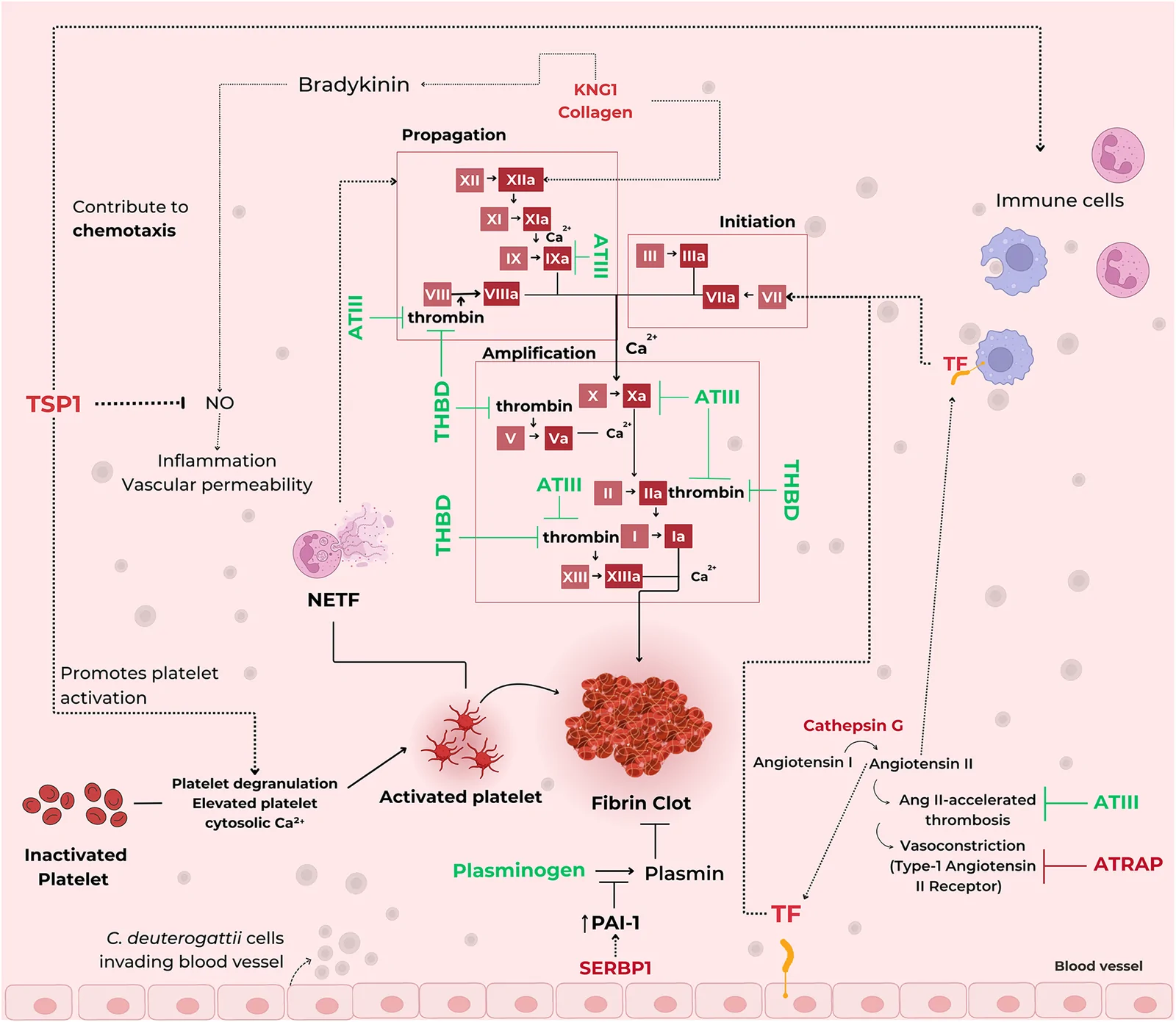
Proposed model for prothrombotic state observed in *C. deuterogattii* infection. The scheme integrates proteomic and functional data, illustrating how the infection promotes platelet activation (associated with elevated cytosolic Ca^2+^ and degranulation, reflecting alterations in pathways such as ’platelet degranulation’ and ’response to elevated platelet cytosolic Ca^2+^’ identified by GSEA), neutrophil extracellular trap formation (NETF), and activation of the coagulation cascade. Dysregulation is evidenced by the exclusive identification of Kininogen-1 (KNG1), Cathepsin G, and type-1 angiotensin II receptor-associated protein (ATRAP) in the 3 dpi group, and by the downregulation of thrombomodulin (THBD) and antithrombin III (ATIII) in infected animals, suggesting perturbation of hemostasis-involved systems. Increased expression of Tissue Factor (TF), which was *in vitro* validated, contributes to fibrin clot formation and the observed prothrombotic phenotype. Red refers to upregulated or exclusively identified, and green to downregulated or not identified.

## Methods

### *Cryptococcus* Culture

50 mL of yeast peptone dextrose (YPD) broth (1% yeast extract, 1% peptone, 2% glucose) was used to grow *C. deuterogattii* strain R265 isolated from the Vancouver outbreak^8^ (18 h, 150 rpm). Cells were harvested by centrifugation (10,000 rpm, 5 min), washed two times with phosphate-buffered saline (PBS), and adjusted to desired cell density by counting in a *Neubauer* chamber.

### Experimental Infection

Adult male Wistar rats (250–300 g) were obtained from the central animal facility of our institution. Animals were housed under standard conditions in a temperature-controlled room (22–23 °C) with a 12 h light/dark cycle (lights on at 7:00 am) and had ad libitum access to water and food. All experimental procedures were conducted according to the Brazilian Federal Law 11.794/2008 and the guidelines of the Brazilian College of Animal Experimentation (COBEA). The study protocol was approved by the Ethical Committee on Research Animal Care of the Federal University of Rio Grande do Sul, Brazil (#17423).

Preliminary tests were conducted to determine the period in which the infection began to establish in the rats, and based on these observations, 3 and 14 days were selected. The 3-day time point corresponds to the stage when the experimental pneumonia model is already established, allowing characterization of the acute phase of infection and inflammation. The 14-day time point represents a prolonged period of disease progression, enabling assessment of late or sustained responses. These two time points were predefined to capture both early and extended effects while minimizing the number of animals used, in accordance with ethical principles for animal experimentation.

Then, for experimental infection, rats (*n* = 6 per group) were anesthetized with intraperitoneal ketamine (75 mg/kg) and xylazine (10 mg/kg). They were divided into: (i) control (no inoculation), (ii) infected intranasally with 0.1 mL of 1 × 10^9^ *C. deuterogattii* R265 cells for analysis at 3 days post-inoculation (3 dpi), and (iii) same inoculation for analysis at 14 dpi. At 3 and 14 dpi, rats were reanesthetized, thoracic cavity opened, and lungs removed. Right lungs were used for histopathology and left lungs for proteomic analysis.

### Histopathological Analysis

Lung tissues, collected as previously described, were fixed in 10% buffered formalin (pH 7.2). Following dehydration through a graded ethanol series and clearing with xylene, samples were embedded in paraffin. Serial sections (4 μm thick) were cut, mounted on slides, and stained with hematoxylin and eosin (H&E).^63^ Histopathological features were examined. All slides were examined by an experienced pathologist in a blinded fashion.

### Coagulation Parameters of Infected Rats

#### Blood Collection and Platelet Counting

Blood was collected from six animals per experimental group (*n* = 6 biological replicates/group) by cardiac puncture under anesthesia and anticoagulated using either (1) 1:5 (v/v) ACD solution (2. 5% trisodium citrate, 1.37% citric acid, 2% d-glucose) for platelet isolation or (2) 1:10 (v/v) 3.8% trisodium citrate. Platelet-rich plasma (PRP) was obtained by centrifugation at 200*g* for 5 min (repeated thrice). Platelet counts were performed manually using optical microscopy in a Neubauer hemocytometer. For counting, a small aliquot of anticoagulated blood was diluted appropriately with 1% ammonium oxalate to lyse red blood cells, allowing clearer visualization and quantification of platelets. The platelet concentration was calculated using the standard formula based on the dilution factor and the volume of the chamber grid. All counts were performed in triplicate for each sample, and the mean values were expressed as the number of platelets per microliter (platelets/μL). Care was taken to avoid sample clotting or platelet clumping, which could interfere with accurate enumeration.

#### Platelet Aggregation Assays

Platelet aggregation was assessed using both turbidimetric and microplate-based methods.^64^ Aggregation and shape change were monitored in PRP suspensions using a Lumi-aggregometer (Chrono-Log Co., Havertown) or a SpectraMax (Molecular Devices Co., Sunnyvale) microplate reader at 650 nm. Aggregation was induced by adenosine diphosphate (ADP, 10 μM) and collagen (2.5 μg/mL). The kinetics of the platelet aggregation process were recorded over 15 min under constant agitation and expressed as area under the curve or changes in turbidity.

#### Blood Coagulation Assays

Platelet-poor plasma was prepared from citrated blood by centrifugation at 1500*g* for 10 min. The following coagulation parameters were measured as previously described:^64^ prothrombin time (PT), activated partial thromboplastin time (aPTT), and recalcification time (RT). All experiments were performed in kinetic mode using a temperature-controlled 96-well microplate spectrophotometer at 650 nm. The changes in the RT assay were recorded following addition of 10 mM CaCl_2_. PT and aPTT tests were assayed in the presence of thromboplastin and ellagic acid, respectively, using commercial kits (Wiener Lab, Argentina).

#### Activity of Blood Coagulation and Fibrinolytic Enzymes

Plasma samples (5 μL) were incubated in 20 mM Tris–HCl buffer (pH 7.4) at 37 °C. Enzymatic activity was measured by adding synthetic chromogenic substrates specific for thrombin (S2238) and plasmin (S2251) at a final concentration of 0.2 mM. The release of p-nitroaniline was monitored at 405 nm every 14 s for 30 min using a SpectraMAX microplate reader. Enzyme activities were expressed as the rate of substrate hydrolysis (mOD/min) and compared to control values (plasma sample from noninfected animals).

### Proteomic Analysis

#### Sample Preparation for Mass Spectrometry

Left lung tissues from six animals per experimental group (*n* = 6 biological replicates/group) were homogenized in cold PBS containing 1% Triton X-100 and protease inhibitors (Halt Protease Inhibitor Cocktail, Thermo Fisher Scientific). The lysates were centrifuged (9500*g*, 15 min, 4 °C), and the supernatants were collected. After lyophilization, the pellets were resuspended in ultrapure water and subjected to methanol/chloroform precipitation. Following drying under vacuum, the resulting protein pellets were reconstituted in 0.1% Rapigest SF (Waters) in 50 mM ammonium bicarbonate (pH 8.0). For each 100 μg aliquot, proteins were reduced with tris­(2-carboxyethyl)­phosphine (TCEP), alkylated with iodoacetamide, and digested overnight with trypsin (Promega, Madison, WI) at 37 °C. Subsequently, trifluoroacetic acid was added to quench the reaction; samples were centrifuged, and the supernatants were stored at −80 °C. Protein concentration was quantified using the BCA assay (Pierce, Rockford, IL).

#### Multidimensional Protein Identification Technology (MudPIT)

The tryptic digest was pressure-loaded onto a 250 μm inner diameter capillary column packed with 2.5 cm of 5 μm Partisphere strong cation exchanger (SCX; Whatman) followed by 2 cm of 3 μm Aqua C18 reversed-phase resin (Phenomenex), which was connected in series to a 11 cm column packed with 3 μm Aqua C18. The entire setup was coupled online to an Agilent 1100 HPLC system and analyzed using a modified 12-step MudPIT protocol as previously described.^18^ The following buffers were employed: Buffer A (5% acetonitrile, 0.1% formic acid), Buffer B (80% acetonitrile, 0.1% formic acid), and Buffer C (500 mM ammonium acetate, 5% acetonitrile, 0.1% formic acid). A total of six independent runs were conducted per treatment group (R265-infected animals at 3 dpi, R265-infected animals at 14 dpi, and uninfected controls). Peptides were directly electrosprayed into an LTQ-XL mass spectrometer (Thermo Fisher Scientific) using a distal spray voltage of 2.4 kV. The acquisition cycle consisted of one full-scan MS spectrum (*m*/*z* 300–2000), followed by five consecutive data-dependent MS/MS scans at a normalized collision energy of 35%. This cycle was continuously repeated throughout each step of the multidimensional separation. To minimize redundant fragmentation of the same peptides, dynamic exclusion was enabled with a repeat count of 1, a repeat duration of 30 s, and an exclusion list size of 200.^65^

#### Protein Identification

Searches were conducted in PatternLab for Proteomics V against the *Rattus norvegicus* proteome (UP000002494_10116), *C. deuterogattii* R265 proteome (UP000029445), and a common contaminants database. Identification criteria followed the PatternLab workflow, using the Comet search engine and SEPro module to estimate false discovery rate (FDR) through a target–decoy strategy, applying a 1% FDR cutoff at both peptide and protein levels. Quantitative analyses were performed using the XIC module, with normalization by total ion current (TIC). Missing values were handled using PatternLab’s imputation procedure, which replaces nondetected features with values near the detection limit. Differential protein abundance was assessed using the TFold and AAPV modules, applying statistical thresholds of *p* ≤ 0.05 and fold change >2, and considering only proteins detected in at least two biological replicates per condition.^66^

#### Proteome Characterization

Protein categorization was performed using DAVID bioinformatics^67^ based on the Gene Ontology for biological processes, molecular function, and cellular components. Results were semantically reduced using REVIGO.^68^ Metabolic pathway analysis followed KEGG.^69^ Gene set enrichment was performed with WebGestalt.^70^ The GO and functional enrichment analyses were performed by considering all proteins from the species as the background list. Networks were built with Cytoscape 3.10.2v^71^ and STRING.^72^ Betweenness and degree analyses used R packages Rcy3 ^73^ and Igraph.^74^ Proteins were manually classified using UNIPROT^75^ on the basis of functional groups, domains, or activities.

### *In Vitro* Experiments

#### Cell Culture

A7R5 (CRL-1444) vascular smooth muscle cells (VSMC) derived from rat thoracic aorta were cultured in Dulbecco’s modified Eagle medium (DMEM) in the presence of 10% fetal bovine serum (37 °C, 5% CO_2_). Cells were passaged at confluence after trypsin/EDTA (0.05%/0.01%) treatment, reseeded into fresh T75 flasks, and used for coculture.

#### Host–Pathogen Interaction Model

To further validate the *in vivo* findings, a coculture system was established using A7R5 cells and *C. deuterogattii* R265. The experimental setup followed the protocol described by Rosa et al.^14^ Briefly, A7R5 cells (5 × 10^3^) were seeded into T75 culture flasks and cultured until confluence. Prior to infection, the growth medium was replaced with serum-free DMEM, and cells were incubated in this condition for 24 h to reduce serum-induced signaling. *C. deuterogattii* R265 was grown in YPD broth at 30 °C for 18 h. Yeast cells were then harvested by centrifugation, washed three times with PBS to remove residual medium, and resuspended in fresh medium. A multiplicity of infection (MOI) of 10 fungal cells per A7R5 cell was added, and cocultures were incubated at 37 °C with 5% CO_2_ for 3, 6, and 12 h. At each time point, the supernatant was discarded, cells were washed three times with PBS, collected, and lysed using RIPA buffer. The resulting lysates were directly analyzed by Western blotting to detect the presence of the target protein (TF).

#### Tissue Factor (TF) Expression

Protein extracts (30–50 μg) from coculture experiments were run on 12% SDS-PAGE under reducing conditions, transferred onto nitrocellulose membranes, incubated with the primary and secondary horseradish peroxidase-conjugated antibodies, and revealed using a chemiluminescent substrate (Bio-Rad, Hercules, CA). The bands were visualized on the iBright Imaging System (Invitrogen, Carlsbad, CA); protein expression levels were normalized against β-actin and quantified using ImageJ software (available at https://imagej.nih.gov/ij/). The following antibody was used for TF detection: tissue factor – 1:500 (I20 #sc-23596 – Santa Cruz Biotechnology, Dallas, TX).

### Statistics Analysis

Data from *in vitro* experiments are expressed as mean ± standard error (SE). Statistical significance between groups was determined using one-way ANOVA. Differences were considered statistically significant at *p* < 0.05. All statistical analyses were conducted using GraphPad Prism software (GraphPad Software, Inc., San Diego, CA).

## Supplementary Material













## Data Availability

The mass spectrometry proteomics data have been deposited in the ProteomeXchange Consortium via the PRIDE partner repository with the data set identifier PXD077765. All data generated or analyzed during this study are included in the Supporting Information available with the online version of this article. Additional data or clarifications may be obtained by contacting the corresponding author.

## Notes

Gene ontology results after semantic reduction (Figure S1) (PDF)
List of identified proteins along with the corresponding statistical data for each experimental condition (Table S1) (XLSX)
List of identified proteins along with the corresponding statistical data for each experimental condition (Table S2) (XLSX)
Summary of the results of the gene ontology analysis (Table S3) (XLSX)
Summary of the results of the gene ontology analysis (Table S4) (XLSX)
Comprehensive data set containing all identified proteins (Table S5) (XLSX)
