# Supplementary material for: Cryptococcus deuterogattii Lung Infection Leads to a Prothrombotic State Revealed by Proteomics and Functional Analysis
Source: ACS Omega. 2026 Oct 28;11(44):68609–20. doi: 10.1021/acsomega.6c07029 (PMC13756680; doi:10.1021/acsomega.6c07029)
Supplement: Supplementary file 1 [file ao6c07029_si_001.pdf]

Cryptococcus deuterogattii lung infection leads to a prothrombotic state revealed by proteomics and functional analysis

Laura Rascovetzki Saciloto-de-Oliveira[1,2,3]; Markus Berger[2]; Luana Cardozo[1,2]; Camila Innocente-Alves[1,2]; David Driemeier[4]; John R. Yates[5]; Marilene Henning Vainstein[6]; Jorge Almeida Guimarães[6]; Lucélia Santi[1,2,6]; Walter Orlando Beys-da-Silva*[1,2,6]

[1] Laboratório de Microbiologia Molecular e Proteômica, Faculdade de Farmácia, Universidade Federal do Rio Grande do Sul (UFRGS), Porto Alegre, RS 90610-000, Brazil;

[2] Faculdade de Farmácia, Universidade Federal do Rio Grande do Sul (UFRGS), Porto Alegre, RS 90610-000, Brazil

[3] Laboratório de Neuroproteômica, Universidade de Campinas (Unicamp), Campinas, São Paulo, Brazil;

[4] Departamento de Patologia Clínica Veterinária, Faculdade de Medicina Veterinária, Universidade Federal do Rio Grande do Sul (UFRGS), Porto Alegre, RS 91540-000, Brazil;

[5] Scripps Research, Department of Molecular Medicine, CA 92037, United States;

[6] Centro de Biotecnologia, Universidade Federal do Rio Grande do Sul (UFRGS), Porto Alegre, RS 90040-060, Brazil;

* Corresponding Author: Pharmacy Faculty, Universidade Federal do Rio Grande do Sul (UFRGS), Porto Alegre, RS 90610-000, Brazil; e-mail: walter.beys@ufrgs.br; phone number: +55513308535

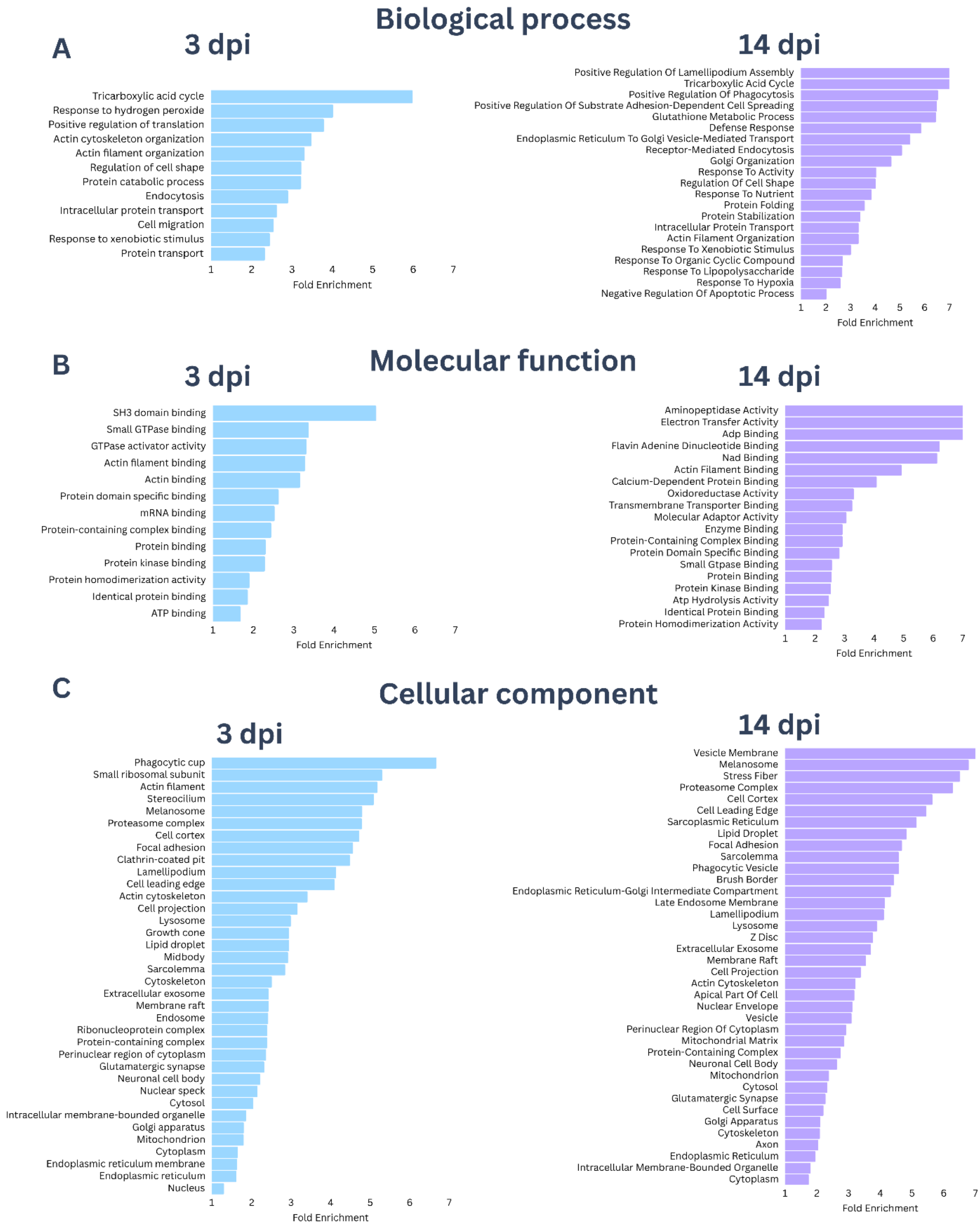

Figure S1. Gene Ontology (GO) analysis of differentially expressed genes in the 3 dpi and 14 dpi groups. The GO categories were grouped into three levels: (A) Biological Processes, (B) Molecular Functions, and (C) Cellular Components. Data were obtained using the DAVID bioinformatics tool, which identified enriched GO terms based on the number of proteins associated with each GO category and calculated the respective fold enrichment. Semantic reduction was performed using the REVIGO tool with the "small" setting, indicating higher stringency in eliminating redundant terms.
